# Whole-genome long-read sequencing downsampling and its effect on variant-calling precision and recall

**DOI:** 10.1101/gr.278070.123

**Published:** 2023-12

**Authors:** William T. Harvey, Peter Ebert, Jana Ebler, Peter A. Audano, Katherine M. Munson, Kendra Hoekzema, David Porubsky, Christine R. Beck, Tobias Marschall, Kiran Garimella, Evan E. Eichler

**Affiliations:** 1 Department of Genome Sciences, University of Washington School of Medicine, Seattle, Washington 98195-5065, USA;; 2 Institute for Medical Biometry and Bioinformatics, Medical Faculty, Heinrich Heine University, 40225 Düsseldorf, Germany;; 3 Core Unit Bioinformatics, Medical Faculty, Heinrich Heine University, 40225 Düsseldorf, Germany;; 4 Center for Digital Medicine, Heinrich Heine University, 40225 Düsseldorf, Germany;; 5 The Jackson Laboratory for Genomic Medicine, Farmington, Connecticut 06032, USA;; 6 Department of Genetics and Genome Sciences, University of Connecticut Health Center, Farmington, Connecticut 06030-6403, USA;; 7 Data Sciences Platform, Broad Institute of MIT and Harvard, Cambridge, Massachusetts 02142, USA;; 8 Howard Hughes Medical Institute, University of Washington, Seattle, Washington 98195, USA

## Abstract

Advances in long-read sequencing (LRS) technologies continue to make whole-genome sequencing more complete, affordable, and accurate. LRS provides significant advantages over short-read sequencing approaches, including phased de novo genome assembly, access to previously excluded genomic regions, and discovery of more complex structural variants (SVs) associated with disease. Limitations remain with respect to cost, scalability, and platform-dependent read accuracy and the tradeoffs between sequence coverage and sensitivity of variant discovery are important experimental considerations for the application of LRS. We compare the genetic variant-calling precision and recall of Oxford Nanopore Technologies (ONT) and Pacific Biosciences (PacBio) HiFi platforms over a range of sequence coverages. For read-based applications, LRS sensitivity begins to plateau around 12-fold coverage with a majority of variants called with reasonable accuracy (F_1_ score above 0.5), and both platforms perform well for SV detection. Genome assembly increases variant-calling precision and recall of SVs and indels in HiFi data sets with HiFi outperforming ONT in quality as measured by the F_1_ score of assembly-based variant call sets. While both technologies continue to evolve, our work offers guidance to design cost-effective experimental strategies that do not compromise on discovering novel biology.

Over the last five years, long-read sequencing (LRS) technologies have transformed the landscape of genetic variant discovery in two fundamental ways. First, they have increased the sensitivity of structural variant (SV) discovery by ∼threefold by providing access to repetitive regions of genomes typically masked or excluded as part of short-read sequencing analyses (Chaisson et al. 2015, 2019; Audano et al. 2019) and by providing breakpoint resolution of variants previously inferred by indirect read-pair or read-depth approaches (Collins et al. 2020). Second, LRS has enabled the routine generation of genome assemblies (Koren et al. 2017; Shafin et al. 2020), and recent advances in sequencing technology and methods are now routinely producing phased genome assemblies fully capturing both haplotypes (Cheng et al. 2021; Porubsky et al. 2021; Lorig-Roach et al. 2023). These advances have begun to improve our understanding of mutational processes, recurrent mutations, and new variants associated with disease and adaptation (Dutta et al. 2019; Begum et al. 2021; Hsieh et al. 2021; Miller et al. 2022; Porubsky et al. 2022).

Consequently, large-scale LRS efforts have enabled the construction of improved reference genomes, including pangenomic representations of species (Liao et al. 2023) and exploration of the pattern of normal and disease variation across a variety of National Institutes of Health (NIH) initiatives in unprecedented detail, for example, the *All of Us* (All of Us Research Program Investigators et al. 2019) and GREGoR (https://www.genome.gov/Funded-Programs-Projects/GREGOR-Consortium, retrieved September 15, 2022) programs. A critical question in such large-scale projects is the tradeoff between sensitivity and specificity for variant discovery as a function of genome coverage. This is especially important given that throughput and cost are still major limitations of LRS. In this study, we attempt to address this issue by comparing two of the most common platforms, Oxford Nanopore Technologies (ONT) and Pacific Biosciences (PacBio) HiFi sequencing, as well as commonly used read-based and assembly-based variant callers. To establish a truth set for comparison, we analyze two deeply sequenced human genomes, HG00733 and HG002, with a specific focus on the recovery of SVs. Realizing that both LRS technologies and variant callers are under continuous development, this analysis is a snapshot in time that aims at informing experimental design to achieve high sensitivity and specificity within realistic economic boundaries.

## Results

Because LRS data can enable phased de novo assembly, we distinguish two LRS approaches for variant discovery: read-based and assembly-based methods. We define read-based methodologies as those requiring alignment of individual sequencing reads to a reference genome and applying specific read-based variant-calling algorithms to these alignments to identify variants. Assembly-based methods, in contrast, first generate ab initio a whole-genome assembly from LRS reads without guidance from a particular reference genome, and then proceed analogously by aligning this assembly to a reference genome to call variants using assembly-based calling algorithms. Many different tools implement variant-calling algorithms and they differ in their support for sequencing technologies (PacBio, ONT, etc.), variant types (SVs, indels, etc.), or data input (assembly, reads, etc.). In this study, we limit our analysis to eight read-based callers (Supplemental Fig. S1): Clair3 [v0.1-r11] (Zheng et al. 2022), cuteSV [v1.0.13] (Jiang et al. 2020), DeepVariant [v1.3.0] (Poplin et al. 2018), DELLY [v1.0.3] (Rausch et al. 2012), PEPPER-Margin-DeepVariant [r0.8] (Shafin et al. 2021), Sniffles [v2.0.2] (Smolka et al. 2022), PBSV [v2.8.0] (https://github.com/PacificBiosciences/pbsv, retrieved April 7, 2003), and SVIM [v1.4.2] (Heller and Vingron 2019), and two assembly-based callers: PAV [v1.2.2] (Ebert et al. 2021) and SVIM-asm [v1.0.2] (Heller and Vingron 2021). Assemblies were generated considering three algorithms: hifiasm [v0.16.1] (Cheng et al. 2021), PGAS [v14-dev] (Ebert et al. 2021; Porubsky et al. 2021), and Flye [v2.9] (Kolmogorov et al. 2019).

We set out to determine how variant-calling performance differs depending on the platform, depth of sequence coverage (×), and computational method. For this assessment, we generated downsampled sets of HiFi and both standard and ultra-long ONT (UL-ONT) sequence data at depths of 5, 8, 10, 12, 15, 17, 20, 25, and 30× assuming a 3.1 Gbp haploid genome size. We applied standard practice algorithms and procedures and evaluated precision and recall of each algorithm for single-nucleotide variants (SNVs), small (<50 bp) indels (insertions and deletions), and SVs with respect to the human reference genome GRCh38. We consider two publicly available human genomes that have been sequenced extensively: HG002 (the Genome in a Bottle [GIAB] Ashkenazim child reference genome) (Wagner et al. 2022a) and HG00733 (a Puerto Rican reference genome from the 1000 Genomes Project). In addition to GIAB analysis of HG002 (Zook et al. 2016), both genomes have been extensively characterized for genetic variants by both the Human Genome Structural Variation Consortium (HGSVC) (Ebert et al. 2021) and Human Pangenome Reference Consortium (HPRC) (Liao et al. 2023), which has led to the availability of thoroughly vetted variant call sets (Ebert et al. 2021) that are used in this study as truth sets (referred to as HGSVC Freeze 4). Both genomes have the advantage that they are targets of telomere-to-telomere (T2T) assembly development (Rautiainen et al. 2023) and, as such, more accurate and complete variant call sets will likely be available in the future to further refine truth sets for comparison. As both of these genomes have been characterized in multiple LRS efforts, sufficiently deep and high-quality input sets are available from both ONT and PacBio. For PacBio HiFi, these sets include 78.6×/17.9 kbp (depth/N50) and 99.54×/20.6 kbp for HG002 and HG00733, respectively. ONT standard length data sets were 153.4×/30.23 kbp and 92.3×/33.6 kbp and the UL-ONT data were 33.15×/96.4 kbp and 38.11×/132.7 kbp for HG002 and HG00733, respectively (Supplemental Table S1).

### Read-based variant calling

**Figure 1.**
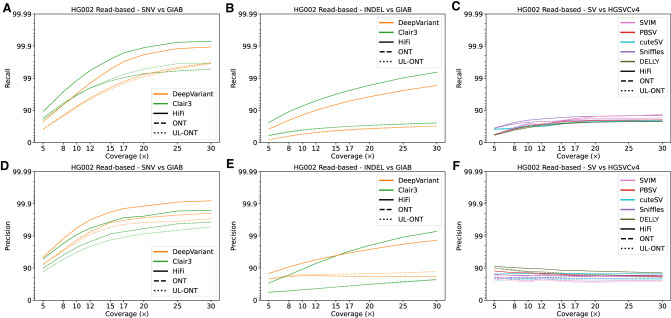
Precision and recall for variant classes as a function of long-read sequencing (LRS) coverage using read-based algorithms for HG002. (*A*) Recall of genome sample HG002 against Genome in a Bottle (GIAB) truth sets plotted against sequencing coverage for read-based callers Clair3 and DeepVariant. Clair3 with PacBio HiFi reaches the earliest recall plateau, whereas all callers show saturation by 20×. (*B*) Recall against GIAB truth sets plotted against sequencing coverage for read-based callers across all algorithms capable of calling indels. Recall of both Clair3 and DeepVariant HiFi sets outperform their ONT counterparts. (*C*) Recall against HGSVC truth sets plotted against sequencing coverage for read-based callers across all algorithms capable of calling structural variants (SVs). (*D*) Precision as a function of sequence coverage. Single-nucleotide variant (SNV) precision remains flat beyond 10×, demonstrating the ability of callers to distinguish sequencing error from true SNVs. (*E*) Precision plotted against sequencing coverage for read-based callers across all algorithms capable of calling indels. Precision values for all technologies and coverages remain flat, but here the increased precision of ONT callers is shown. (*F*) Precision plotted against sequencing coverage for read-based callers across all algorithms capable of calling SVs.

Read-based SNVs were called with DeepVariant and Clair3 and showed the least variability between callers and technologies out of all three variant categories. At sequence read depth below 15×, recall of PacBio HiFi-tuned algorithms consistently outperformed ONT by an average of 0.06 (Fig. 1A,D). In fact, at ∼10× coverage (current production from a single Sequel II SMRT cell) both precision and recall for HiFi data plateau while reaching a precision of 0.99 and recall of 0.98. At 5× coverage, DeepVariant and Clair3 showed on average 0.09 higher F_1_ scores in PacBio compared to ONT (Supplemental Table S2). This was shown in both precision and recall with DeepVariant performing better with respect to precision and Clair3 with respect to recall. At coverage depths above 15×, the F_1_ score plateaued around 0.96 with recall being consistently higher than precision for all callers and technologies. The data suggest that HiFi is generally better with regard to recall but that 12× standard ONT and HiFi perform comparably. When evaluating SNV accuracy in a second sample, HG00733, against the HGSVC Freeze 4 data set (Supplemental Fig. S2), we notice that these trends hold albeit with slightly depressed values because of the nature of the SNV calling in that effort compared to the GIAB data sets. SNV calling for HG002 performed by GIAB has been subjected to extensive QC and specific regions are likely under called. In our analysis of 30× coverage data sets, we observed 13,147 SNV calls not seen in GIAB for HG002. Of these 13,147 calls, 324 (2.46%) were observed by all three technologies using DeepVariant. These SNVs, in addition to those meeting the same criteria with assembly-based callers, are included in the Supplemental Material (Supplemental Fig. S3; Supplemental Table S3) and are proposed as potential variants for inclusion in future GIAB releases.

Indels, defined here as insertions or deletions <50 bp, show a similar profile. There is, once again, a characteristic plateau in F_1_ score around 12× sequence coverage in HiFi sequencing data; however, this occurs with an F_1_ score of 0.65. The ONT F_1_ score plateaus around 20× at 0.56. The greatest difference in recall is shown in this subset between the HiFi and ONT platforms (based on the R9 nanopore technology) (Fig. 1B,E). While precision remains comparable between ONT and HiFi parameterizations of DeepVariant and Clair3 with an average of 0.54 across all measured depths, recall is noticeably lower in ONT when compared to PacBio HiFi reads, on average 0.28 less at depths less than or equal to 12× and 0.35 above 12× (Supplemental Table S4). For this class of variant, ONT reads prepared with standard library prep perform in line with their UL-ONT counterparts with respect to precision and recall. Overall, recall for indels is higher in HiFi data sets at all coverages, whereas ONT callers are comparably precise. A large amount of community development has gone into refining variant callers for ONT and has allowed these call sets to reduce noise inherent to less accurate ONT sequence reads at the cost of lower discovery rates.

**Figure 2.**
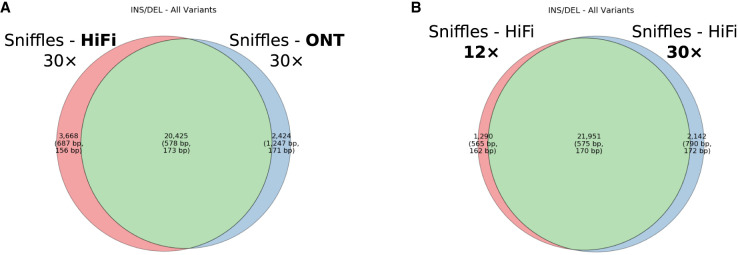
SV discovery. (*A*) Venn diagram comparing Sniffles detection of SVs (both insertions and deletions) for 30× HiFi and 30× standard ONT input read sets. (*B*) Venn diagram comparing Sniffles SV discovery at 12× and 30× HiFi call sets. A consistent set of calls is generated above 12×.

For SVs, we consider only insertions and deletions greater than or equal to 50 bp and annotated with QUAL >10. SVs show the least variability between technologies (Fig. 1C,F) (F_1_ standard deviation of 0.01 between HiFi and ONT sequencing platforms [Supplemental Table S5]). Both sequencing platforms and various coverages converge on a set of ∼12,800 SVs with each calling on average 25,634 SVs (Fig. 2A). Of the variants unique to one technology or the other, 85% map to tandem repeat regions, which suggest breakpoint resolution rather than technology-specific bias driving the difference. Different read-based callers, however, show considerable variation. While recall remains low at lower sequence depth (Fig. 2B), mainly because of random sampling bias, two callers stand out as having the greatest precision: PBSV and DELLY. Both callers consistently perform with high precision (mean 0.89) at low coverage depths and remain consistently high as depth increases. However, this does come with the above-mentioned tradeoff between precision and recall. As one increases, the other will decrease. Because of a static quality score cutoff, this relationship may not remain universally true, as different quality thresholds reduce this tradeoff (Supplemental Fig. S4). In terms of recall at low-coverage sequence read depths below 12×, Sniffles performs best with a mean 0.63/0.84/0.71 precision/recall/F_1_ with cuteSV a close second (0.57/0.84/0.67). These estimates of precision and recall are based solely on the detection of the alternate SV allele.

### Assembly-based variant calling

Assembly-based callers have the advantage that they call variants from large contiguous haplotype blocks essentially providing access to larger and more complex forms of genetic variation and providing extended phasing for all forms of genetic variation (Wagner et al. 2022b). We generated assemblies using three algorithms: hifiasm (v0.16.1), PGAS (v14-dev), and Flye (v2.9) where applicable. Hifiasm and PGAS assemblies were generated for the PacBio HiFi readsets, and Flye assemblies for the ONT reads. All variants were called using the phased assembly variant (PAV) caller (Ebert et al. 2021) in addition to SVIM-asm specifically for SVs. The state of genome assembly for HiFi and ONT are not easily comparable; whereas HiFi reads can be assembled with numerous algorithms and assessed for phasing accuracy, ONT reads provide a greater challenge because of higher sequence error and fewer algorithms that combine both assembly and phasing. Methods such as Shasta (Shafin et al. 2020), wtdbg2 (Ruan and Li 2020), and Canu (Koren et al. 2017) show considerable promise, yet currently contiguous, haplotype-phased assemblies are not as easily generated and thus have not been used as frequently in recent studies.

**Figure 3.**
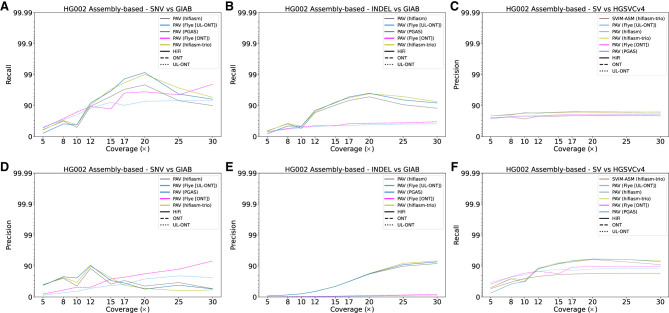
Precision and recall for variant classes as a function of LRS coverage using assembly-based algorithms for HG002. (*A*) Recall for HG002 for GIAB truth sets plotted against sequencing coverage for assembly-based callers across all algorithms capable of calling SNVs. (*B*) Recall for HG002 against HGSVC truth sets plotted against sequencing coverage for assembly-based callers across all algorithms capable of calling indels. Recall in ONT assemblies performs better at low coverages before being surpassed by HiFi assemblies at 12×. (*C*) Recall for HG002 against the HGSVC Freeze 4 truth set plotted against sequencing coverage for assembly-based callers across all algorithms capable of calling SVs. (*D*) Precision for HG002 against HGSVC truth sets plotted against sequencing coverage for read-based callers across all algorithms capable of calling SNVs. ONT methods are comparable to HiFi precision at high coverages though are noticeably worse at coverages below 15×. (*E*) Precision plotted against sequencing coverage for assembly-based callers across all algorithms capable of calling indels. Like read-based methods, values for all technologies and coverages remain low, likely because of the incomplete nature of indels in complex regions in the GIAB truth set. (*F*) Precision plotted against sequencing coverage for assembly-based callers across all algorithms capable of calling SVs.

**Figure 4.**
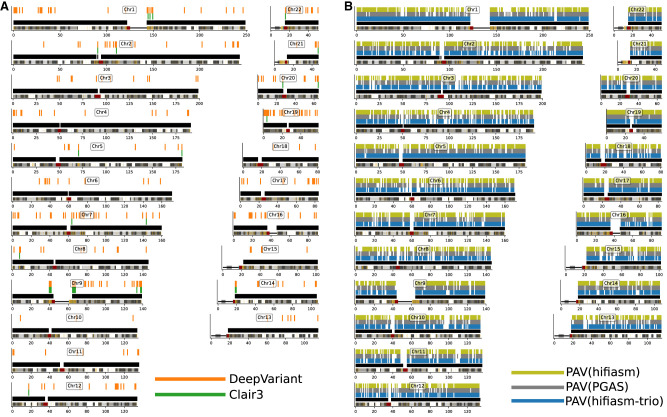
Ideogram comparison of autosomal SNV recall at 8× for PacBio HiFi. (*A*) PacBio HiFi (8×) read-based recall of HG002 SNVs against GIAB truth sets. A bar *over* a chromosome depicts a 1 Mbp window where there was <90% SNV recall for Clair3 (green) and DeepVariant (orange) with all regions where SNVs were called in black. Highlighted regions represent limitations in methodology at low coverage. (*B*) PacBio HiFi (8×) assembly-based recall of HG002 GIAB SNV truth set using PAV. There are more 1 Mbp windows with <90% recall irrespective of assembly algorithm including hifiasm-trio (yellow), PGAS (blue), or hifiasm (gray).

SNV calling with assembly-based callers generally underperforms read-based discovery especially at lower coverages. Precision in ONT and UL-ONT assembly-based methods shows the greatest difference with an average reduction of 0.38 across all sequencing depths (Fig. 3A,D). This is especially true in low-coverage (<12×) scenarios and is driven by an excess of assembly-based SNV calls in ONT data sets (mean 8.33M in ONT; mean 10.00M in UL-ONT). PacBio HiFi methods have the opposite problem in that they underreport SNVs with a mean of 3.00M calls, although that does not greatly affect precision. This under calling in HiFi assembly-based SNV call sets is a result of far less of the genome being assembled into haplotype-resolved contigs at lower coverages (Fig. 4B). The main effect on precision is because of genotyping errors (Supplemental Fig. S5), which are much more common in assembly-based methods compared to read-based methods. However, when coverage reaches 12×, assembly-based methods show excellent recall (mean 0.94) for SNVs across all technologies (Supplemental Table S6), which mirrors the plateau observed in read-based methods. Below this threshold, read-based callers recall nearly 4× more (2551 vs. 651) SNV windows based on recovery of over 90% of variants partitioned into 1 Mbp (Fig. 4A). Overall, SNV calling in low-coverage (less than 12×) assemblies is not recommended, but coverages at or above 12× provide comparable precision as their read-based counterparts with an average of 0.04 lower recall or a percent increase of 798%.

Detecting indels from assembly-based methods is especially challenging (Fig. 3B,E), in part because of the known LRS error profiles associated with indels of smaller motif sizes (Wenger et al. 2019; Delahaye and Nicolas 2021). Inability to correct these errors at low sequencing depth significantly inflates indel counts (1,145,880 indel insertion calls on average in PacBio HiFi 5× vs. 444,045 indel insertion calls in PacBio 30×). As such, precision is lowest for indels called in assemblies below 12× (Supplemental Table S7). In ONT data sets, this issue is exacerbated by an order of magnitude at reduced coverages (8,105,758 at 5×) and remains problematic even at high coverage (1,137,763 at 30×). Precision estimates, however, may be underestimated because of the limited capability of Illumina to detect variation in more complex regions of the genome that were not accessible to the GIAB truth set. Additional development and orthogonal validation of indels should be an active area of LRS technology development.

SVs follow the trend of assembly-based call sets in general with a steep recall curve, steady precision curve, and early plateau across sequencing depths and technologies (Fig. 3C,F). For low (below 8×) HiFi coverages, assembly-based methods underperform their read-based counterparts with respect to recall by an average of 0.03 (Supplemental Table S8). ONT assemblies show higher recall than their read-based counterparts by 0.09 and 0.10 for standard ONT and UL-ONT, respectively. Above this coverage, all assembly-based methods outperform read-based methods by at least 0.08 for recall. The HG002 assemblies using PacBio HiFi reads at 10× sequencing depth are a clear outlier and may be attributable to a systematic failure to remove false duplications, which can affect variant calling in all variant classes. PAV is especially susceptible to false duplications impacting recall because of its alignment trimming algorithm. While less pronounced, we did observe a similar outlier in HG00733 (Supplemental Fig. S6). Although the assembly size for HG002 is larger than expected, metrics such as contiguity (N50) and callable loci are consistent with other assemblies. Similar outliers may be avoidable with deeper coverage to support high-quality assembly-based call sets (Ebert et al. 2021; Liao et al. 2023).

### Cross-call-set comparisons

**Figure 5.**
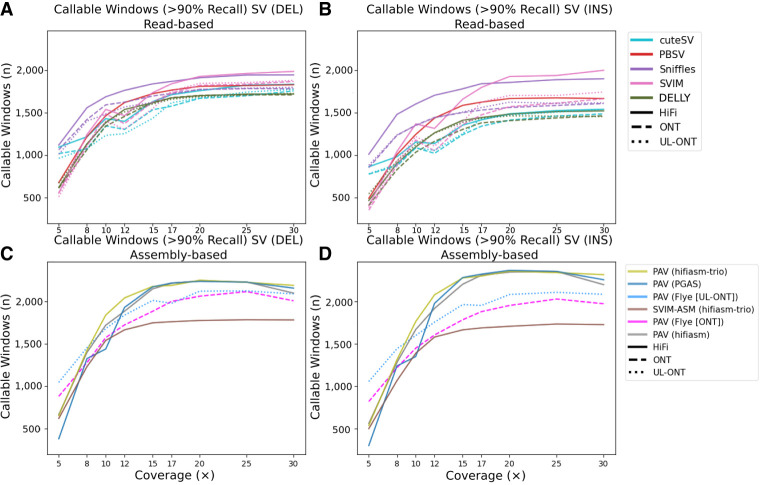
Evaluation of SV callable bases by technology and algorithm. Read-based callable windows for (*A*) deletions and (*B*) insertions, and assembly-based callable regions for (*C*) deletions and (*D*) insertions. Regions were compared against the HGSVC HG00733 truth set in 1 Mbp windows requiring at least 90% recall.

Because LRS technologies claim to access more of the genome and more complex classes of genetic variants, we first evaluate genome-wide SV callability. To assess callability across the genome, we first divided GRCh38 into 1 Mbp windows and intersected those windows with the HGSVC SV truth set for HG00733, yielding 2679 and 2482 windows for insertions and deletions, respectively. While this only represents 84% of the genome, in this analysis we are only considering windows here where an SV was identified and if we consider all 1 Mbp windows where sequence could be evaluated this rises to 92%. A similar comparison to regions accessible with short-read sequencing technologies recovers only 85% (Wagner et al. 2021). In order for a window to be established as callable, >90% of the calls contained in this window must be accurately recovered (Fig. 5A–D). At low coverages (5×), read-based methods outperform assembly-based methods for each respective technology. At these low coverages, Sniffles used with HiFi reads performs the best, recovering 1118/2482 (45%) windows when considering deletion calls. This is almost double the PacBio HiFi callable windows for assembly-based methods. This trend holds for insertions, but we do note that Flye assembly-based methods using UL-ONT perform better than Sniffles on HiFi reads. At 10× and above, the pattern switches with HiFi assembly methods outperforming all read-based callers with the starkest difference occurring at 15× where assembly-based methods recover an additional 500 Mbp and 383 Mbp of the genome for insertions and deletions, respectively, than read-based methods.

### SVs in clinically important genes in HG002

A list of SVs for clinically relevant genes was released for the GIAB sample HG002 (Wagner et al. 2021), including 273 challenging genes or regions that map to repetitive and structurally complex polymorphic regions. At 30× coverage, PBSV was able to recover 97% of these SVs in clinically relevant genes (Supplemental Table S9). However, at the lowest coverage depths, Sniffles, once again, drastically outperformed the other callers across all technology types, but especially with PacBio HiFi reads where it reports recall of 0.87 and 0.82 for SV insertions and deletions, respectively, at just 8× sequencing coverage. Compared to read-based methods, assembly-based methods showed lower recall at low coverages with a max of 0.72 for insertions and 0.79 for deletions using Flye with UL-ONT and hifiasm (nontrio binned), respectively (Supplemental Table S10).

### Tandem repeat characterization

LRS technologies allow for more robust characterization of tandem repeats (Chaisson et al. 2015; Pendleton et al. 2015; Sedlazeck et al. 2018; Chaisson et al. 2019), the largest of which are known as variable number of tandem repeats (or VNTRs). After SNVs, tandem repeat variants are among the most abundant forms of human genetic variation comprising >20% of indels and >50% of SVs (Supplemental Table S11; Ebert et al. 2021). Excluding these regions from analysis has little effect on recall, indicating that even though these regions have been difficult to characterize in prior studies, most LRS technologies and algorithms are able to detect these variants despite ambiguity in defining the exact breakpoints. However, inclusion of these regions potentially comes with a tradeoff in precision, particularly with read-based methods. To evaluate this, the ratio of log_10_(1-Precision) was compared in read- and assembly-based methods (Supplemental Fig. S7) revealing a mean ratio of 1.78 (TR/NOTR) in read-based methods. Assembly-based methods were less affected by these regions with a precision ratio near 1 (0.98). This indicates that even at low coverages assembly-driven variant calling can characterize such variation.

### Performance in homopolymer DNA

Accurately calling variants in homopolymer runs is challenging for both PacBio HiFi and ONT applications (Logsdon et al. 2020; Shafin et al. 2021; Mc Cartney et al. 2022). These nonrandom error profiles impact precision and recall, especially for indel variant calls. When comparing the difference between all indel calls annotated with and without homopolymers, ONT call sets display a large difference between homopolymer and non-homopolymer DNA sequence precision and recall (Supplemental Fig. S8). Even at high coverages, recall for insertions in homopolymer sequence is as much as 0.13 lower than when compared against the whole set. The effect that these sequence types have on precision even at higher depths is still prevalent with even 30× read-based methods showing a decrease of 0.09 between these regions. DeepVariant calls for UL-ONT reads show a decrease in homopolymer precision as sequencing depth increases. This could be because of a prior lack of training data with a ground truth for complex genomic regions uniquely aligned by this technology.

### Genotyping accuracy

Comparison of reported genotypes in SNVs and indels reveal a high error rate in assembly-based methods compared to read-based methods. Assembly-based methods, on average, show a greater than fourfold genotyping error rate in indels compared to read-based methods, and a >17-fold difference with regard to SNVs (Supplemental Fig. S5). This observation can be driven by two main factors: assembly accuracy and caller optimization. Especially at lower coverages, assemblies are prone to false homozygosity driven by a lack of reads affecting the assembly graph. In addition to this, PAV is primarily designed for larger variants and not tuned to capture some classes of SNVs, which results in the genotyping error rate remaining high even at high coverage.

### Large variant discovery

Large (>10 kbp) SVs, especially insertions within or near repeat regions, frequently evade Illumina detection (Medvedev et al. 2009). An advantage of LRS technologies is that these events can be detected directly from the sequence of the reads or the assembly themselves. We assessed each method's ability to recover large variants using the HGSVC validation set from HG00733, including 63 deletions and 40 insertions. For HiFi reads, two trends emerge: their limitation in detecting large insertions compared to ONT reads, likely because of increased ONT sequence read length, and their increased recall when assembled even at low coverages. HiFi reads consistently lag behind their ONT counterparts for large insertions, recovering only half of the insertions in standard ONT call sets and a third of the insertions detected in UL-ONT (Supplemental Table S12). However, by assembling these reads, HiFi data sets outperform ONT when sequence coverage exceeds 8×. Among read-based methods, UL-ONT performs the best with a minimum of 21/63 large deletions and 15/40 large insertions detected even at low sequence coverages (5×). Across all read-based algorithms, Sniffles recovers the greatest number of large events with a maximum of 0.67 and mean of 0.51 recall over all input types and coverages followed by cuteSV with 0.65 and 0.41, respectively. It should be noted that DELLY failed to call any SVs above 10 kbp. HiFi assembly-driven methods perform the best overall with a maximum large variant recall of 0.87 and a mean of 0.65 when PAV is used (Supplemental Table S13). Finally, both read- and assembly-based methods recovered the largest (238 kbp) deletion, but only assembly-based methods identify the largest insertion of 51 kbp compared to the maximum event size in read-based methods of 32 kbp.

### ONT duplex reads and Revio HiFi data

**Figure 6.**
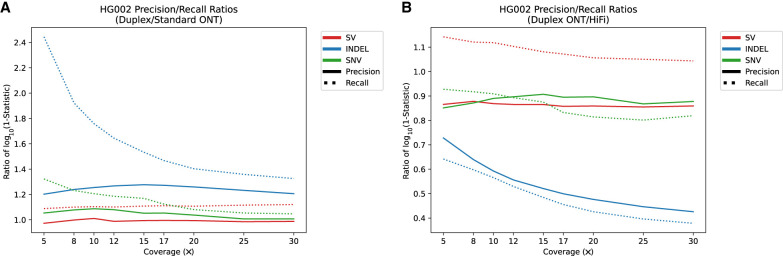
Comparison of precision and recall in duplex ONT variant calling versus standard ONT and HiFi. (*A*) Ratio of log_10_(1-Statistic) where Statistic is either precision (solid line) or recall (dotted line) of duplex ONT compared to standard ONT sequencing. Anything *above* the y = 1 line indicates an increase in performance compared to standard ONT and anything *below* the y = 1 line indicates a decrease in performance compared to standard ONT. (*B*) Ratio of log_10_(1-Statistic) where Statistic is either precision (solid line) or recall (dotted line) of duplex ONT compared to HiFi sequencing. Anything *above* the y = 1 line indicates an increase in performance compared to standard ONT and anything *below* the y = 1 line indicates a decrease in performance compared to HiFi.

PacBio and ONT are rapidly developing new sequencing technologies that improve LRS accuracy and throughput. For example, ONT recently released an improved flowcell (R10) as well as a new “duplex” sequencing method (https://nanoporetech.com/about-us/news/oxford-nanopore-tech-update-new-duplex-method-q30-nanopore-single-molecule-reads-0, retrieved April 8, 2023) significantly improving individual read accuracy by sequencing both forward and complementary strands from the same single molecule (Sanderson et al. 2023). The new release of the Revio system from PacBio, in contrast, significantly increases throughput and affordability using a chemistry similar to that of the Sequel II platform (i.e., HiFi sequencing). The recent release of whole-genome sequencing (WGS) data sets from the GIAB sample HG002 allows these new emerging LRS platforms to be compared. We analyzed a 30× duplex data set of WGS data released by ONT and compared precision and recall to standard ONT using R9.4.1 flowcells. We find that variant-calling recall for specific variant classes is substantially improved for duplex sequencing over R9 ONT variant calling at all sequence coverages and for all variant classes. The effect is most pronounced for indel recall at low coverage (≤10×) where duplex variant recall improves by 0.19 (Fig. 6A,B) when compared to standard ONT. Precision, however, is much more consistent with standard ONT methods. Of note, in our analysis, the precision of indel insertions actually diminishes when compared to standard ONT (an average of 0.06 reduction). This is possibly because of parameterization of variant-calling algorithms, which have been largely adjusted for calling in a noisier, more error-prone, single-strand ONT signal.

Using 30× of WGS data from HG002 generated by the Revio system (https://www.pacb.com/revio/, retrieved October 26, 2022), we also constructed a phased human genome assembly using hifiasm. The results were nearly identical to an assembly produced from a Sequel II HiFi data set, albeit with a single flowcell. Both the contiguity (contig N50 = 44 Mbp [Revio] vs. 45 Mbp [Sequel II]) and accuracy based on quality value (QV) (57 [Revio] vs. 55 [Sequel II]) were virtually identical. Predictably, assembly-based variant calling was comparable for both recall (Pearson R = 0.984) and precision (Pearson R = 0.997) with some modest improvements in SNV recall (+0.02 vs. both truth sets) and small insertion precision (+0.06 vs. HGSVC Freeze 4) (Table 1).

**Table 1.**
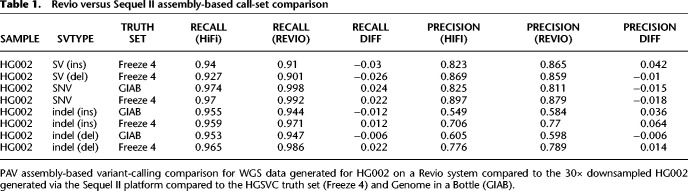
Revio versus Sequel II assembly-based call-set comparison

## Discussion

Within the limits of the various algorithms and sequencing platforms analyzed here, we make a few general observations and recommendations based on our analysis against current truth sets (Zook et al. 2016; Ebert et al. 2021). With respect to SNV discovery, LRS coverage in excess of 12-fold begins to show a plateau with respect to sensitivity. Read-based approaches such as Clair3 (Zheng et al. 2022) and DeepVariant (Poplin et al. 2018) significantly outperform assembly-based detection methods, such as PAV, which have been geared to improve SV discovery and breakpoint definition (Audano et al. 2019; Ebert et al. 2021). While Clair3 with PacBio HiFi performs the best for SNVs, both deep convolutional network approaches (Clair3 and DeepVariant) show excellent recall with both ONT and PacBio above 20× sequence. Irrespective of the sequencing platform, sequence coverage at 8× or lower shows significant reduction in performance and is not advised for large-scale sequencing projects dedicated to variant discovery.

By contrast, all LRS platforms currently underperform for indel variant calling and, predictably, they perform the most poorly in regions of homopolymer runs as well as short tandem repeats—precisely the regions that are most mutable for this class of variation (Willems et al. 2014). Given that caveat, we would recommend PacBio HiFi read-based methods for recall across all read coverages and ONT for precision, although the difference is slight and can be tweaked by filtering out variants using additional metrics such as GQ or QUAL. A major challenge facing human genetics is the existence of a well-vetted and complete truth set for indel variants—detailed studies over the years have restricted analyses to specific regions of the genome owing to the high rate of false positives and false negatives from more mutable and difficult-to-sequence regions (Krusche et al. 2019; Zook et al. 2019; Olson et al. 2022). Our results suggest that haplotype-resolved assemblies offer some improvement for recall. Completely sequenced and assembled genomes where T2T chromosomal assemblies are established along with vetted indel call sets by multiple sequencing technologies (e.g., Sanger, Illumina, ONT, and PacBio) will be required to develop a more comprehensive truth set of indels for benchmarking. Resources such as the Platinum pedigree (CEPH pedigree 1463) by Illumina will be particularly useful as they enable studying phased genome assemblies and variant calling in the context of transmission within families (Eberle et al. 2017).

Both PacBio HiFi and ONT excel at SV detection, routinely detecting >20,000 SVs and consistently calling the same variants when sequence coverage exceeds 12× (Fig. 3). SVs that are unique to one platform over another map to tandem repeat regions but are in close proximity (<10 kbp) to variants called by other technologies and their size overlap suggests that differences in alignment and breakpoint definition are still potentially more rate-limiting as opposed to platform differences in sensitivity. The advance of LRS for SV detection when compared to Illumina WGS has been well established over the years (Chaisson et al. 2015, 2019; Sedlazeck et al. 2018; Shafin et al. 2021) and more sophisticated callers as well as computational pipelines continue to be developed to discover and characterize SVs as part of routine call sets (Kolmogorov et al. 2023). ONT, and especially UL-ONT, performed well for detecting large insertions (Supplemental Table S12) and the advantage here is driven primarily by larger read lengths that can more often traverse large repetitive DNA to anchor alignments in unique flanking sequence. Overall, assembly-based approaches (especially hifiasm) showed the greatest specificity and precision when calling large SVs (>50 kbp) (Supplemental Table S13). Because large SVs are much more likely to have phenotypic consequence and precise breakpoints are relevant to the effect of this consequence, assembly-based strategies should strongly be considered when applying LRS to solving cases of Mendelian and de novo disease (Miller et al. 2021). However, the generation of phased genome assemblies requires deeper sequencing coverage (at least 15–20×) and, as such, is still a more expensive option (Supplemental Figs. S9–S12; Supplemental Analyses). Such deeper data sets have the added advantage of improving long-range phasing accuracy (Supplemental Fig. S11; Supplemental Analyses) and integrating CpG methylation with haplotypes leading to better interpretation of the clinical significance of pathogenic mutations (Miller et al. 2022). There are, thus, considerations other than improved variant detection for choosing LRS.

In summary, when deciding LRS depth targets, the intended purpose of the project must be considered. If the goal is recovery and characterization of SNVs at a population scale, low-depth read-based methods will provide the right balance of maximizing discovery and number of samples in the study. However, if the goal is sequence resolution of large and complex variants at the level of individual patients, assembly-based methods, in particular hifiasm, are currently one of the most accurate strategies for building phased genome assemblies though these require greater investment in terms of sequence coverage (well beyond 15×) and computational processing. The LRS platforms continue to rapidly evolve in terms of accuracy (ONT) and throughput (PacBio). Improved modeling of the platform-dependent errors as well as newer pores or techniques (duplex sequencing) for ONT show considerable promise with suggestions that variant detection accuracy may in fact rival or surpass that of Illumina (Kolmogorov et al. 2023). Changes, such as duplex sequencing with the R10 pore, however, currently come at a cost of lower throughput (Sanderson et al. 2023) and, as a result, added expense to achieve deep coverage. For the last three years, PacBio HiFi has dominated the field with respect to accuracy in large part because of the advent of circular consensus sequencing (CCS); however, multiple flowcells have been required to achieve deep sequence. The release of the new Revio platform earlier this year significantly increases throughput and decreases costs, which will aid production of high-quality and contiguous assemblies comparable to that of those generated previously by multiple Sequel II flowcells. Both platforms are currently highly complementary. Recently, algorithms that aim to incorporate the strengths of both PacBio HiFi and ONT reads to generate de novo T2T assemblies have shown very promising results (Rautiainen et al. 2023). Such hybrid technology approaches have the potential to supplant any single LRS technology as soon as the costs drop and the production of LRS assemblies become routine. The benefit of complete T2T variant discovery should not be underestimated.

## Methods

### ONT data generation

UL-ONT data were generated from the HG00733 lymphoblastoid cell line according to a previously published protocol (Logsdon 2022). Briefly, 3–5 × 10^7^ cells were lysed in a buffer containing 10 mM Tris-Cl (pH 8.0), 0.1 M EDTA (pH 8.0), 0.5% w/v SDS, and 20 µg/mL RNase A (Qiagen, 19101) for 1 h at 37°C. Next, 200 µg/mL Proteinase K (Qiagen, 19131) was added, and the solution was incubated at 50°C for 2 h. DNA was purified via two rounds of 25:24:1 phenol-chloroform-isoamyl alcohol extraction followed by ethanol precipitation. Precipitated DNA was solubilized in 10 mM Tris (pH 8.0) containing 0.02% Triton X-100 at 4°C for 2 d. Libraries were constructed using the Ultra-Long DNA Sequencing Kit (ONT, SQK-ULK001) with modifications to the manufacturer's protocol. Specifically, ∼40 µg of DNA was mixed with FRA enzyme and FDB buffer as described in the protocol and incubated for 5 min at RT, followed by a 5-min heat inactivation at 75°C. RAP enzyme was mixed with the DNA solution and incubated at RT for 1 h before the clean-up step. Clean-up was performed using the Nanobind UL Library Prep Kit (Circulomics, NB-900-601-01) and eluted in 225 µL EB. Finally, 75 µL of library was loaded onto a primed FLO-PRO002 R9.4.1 flowcell for sequencing on the PromethION, with two nuclease washes and reloads after 24 and 48 h of sequencing.

### PacBio HiFi data generation

PacBio HiFi data were generated from the HG00733 lymphoblastoid cell line as previously described (Logsdon et al. 2021) with modifications. Briefly, DNA was extracted from 4.3 × 10^6^ cells using the Monarch HMW DNA Extraction Kit for Cells and Blood (New England Biolabs) with 1400 rpm lysis speed. After UV absorption and fluorometric quantification (Qubit High Sensitivity DNA kit, Thermo Fisher Scientific) on the DS-11 FX instrument (Denovix) and evaluation of DNA integrity on FEMTO Pulse (Agilent), 12 μg of DNA was prepared for sequencing using Megaruptor 3 shearing (Diagenode, settings 19/31) and the Express Template Prep Kit v2 and SMRTbell Cleanup Kit v2 (PacBio). The library was size-selected on a PippinHT instrument (Sage Science) using a 15 kbp high-pass cut. Five SMRT Cell 8Ms were run on a Sequel II instrument using Sequel II chemistry C2.0/P2.2 with 30-h movie times, 2-h pre-extension, and adaptive loading targets of 0.8–0.85 (PacBio). Circular consensus calling was performed with CCS version 6.0.0 (SMRT Link v.10.1) and reads with estimated quality scores ≥Q20 were selected for downstream analysis.

### External data sets

HG002 HiFi data were acquired as part of the HPRC and are available at this s3 address: s3://human-pangenomics/T2T/ scratch/HG002/sequencing/hifi/. HG002 ONT, UL-ONT, and duplex ONT data were acquired from the EPI2ME project (https://epi2me.nanoporetech.com/, retrieved April 25, 2023) and are available in this s3 bucket: s3://ont-open-data/. HG002 Revio data were acquired directly from PacBio and are available here: https://downloads.pacbcloud.com/public/revio/2022Q4/.

### Comparison sets

Genome in a Bottle (GIAB) v4.2.1 was used to compare SNVs and indels in HG002. The VCF is available for download here: https://ftp-trace.ncbi.nlm.nih.gov/ReferenceSamples/giab/release/AshkenazimTrio/HG002_NA24385_son/NISTv4.2.1/GRCh38/. HGSVC Freeze 4 VCFs, which were used to compare all variant types in both HG00733 and HG002, are available here: http://ftp.1000genomes.ebi.ac.uk/vol1/ftp/data_collections/HGSVC2/release/v2.0/integrated_callset/.

### Reference genome and reliable regions

To support long-read mapping, only the primary GRCh38 assembly was used, which includes chromosome scaffolds, the mitochondrial assembly, unplaced contigs, and unlocalized contigs. No alts, patches, or decoys were present in the assembly during the alignment stages. This reference was used previously (Audano et al. 2019; Ebert et al. 2021) and is available for download here: http://ftp.1000genomes.ebi.ac.uk/vol1/ftp/data_collections/HGSVC2/technical/reference/20200513_hg38_NoALT/. Whole-genome analysis was restricted to regions outside centromeres, pericentromeric repeats, and the mitochondrial chromosome where variant calls were previously determined to be less reproducible (Audano et al. 2019; Ebert et al. 2021). This is available here: http://ftp.1000genomes.ebi.ac.uk/vol1/ftp/data_collections/HGSVC2/technical/filter/20210127_LowConfidenceFilter/.

### Downsampling

In-house Python scripts (Supplemental Code) were used to read indexes for our input data sets and subsample reads randomly up to the desired threshold. We then used SAMtools (Danecek et al. 2021) fqidx to extract the desired reads from our larger sets and partitioned them into individual bins.

### Whole-genome alignment

ONT and PacBio reads were aligned with minimap2 v2.21 (Li 2018). Specific commands used can be referenced in the Supplemental Material.

### Assemblies

We used two approaches to generate phased whole-genome assemblies for all PacBio HiFi sampling depths: we used the PGAS pipeline as previously described (parameter settings v14-dev) (Ebert et al. 2021; Porubsky et al. 2021), which does not rely on parental data to derive genome-wide phase information. Additionally, we executed hifiasm v0.16.1 (Cheng et al. 2021) with default parameters in trio-binning mode, leveraging parental short reads to obtain phase information. For the ONT and UL-ONT read sets, we implemented a two-step process using first the Flye assembler v2.9 (Kolmogorov et al. 2019) to generate unphased whole-genome assemblies with default parameters (preset “‐‐nano-hq” and “‐‐genome-size” of 3.1 Gbp). Next, these assemblies were converted into diploid assemblies using the HapDup v0.6 tool (Kolmogorov et al. 2019; Shafin et al. 2020) with default parameters (preset “ont”).

### Read-based variant calling

We used Clair3 [v0.1-r11] (Zheng et al. 2022), cuteSV [v1.0.13] (Jiang et al. 2020), DeepVariant [v1.3.0] (Poplin et al. 2018), DELLY [v1.0.3] (Rausch et al. 2012), PBSV [v2.8.0] (https://github.com/PacificBiosciences/pbsv, retrieved April 7, 2003), PEPPER-Margin-DeepVariant [r0.8] (Shafin et al. 2021), Sniffles2 [v2.0.2] (Smolka et al. 2022), and SVIM [v1.4.2] (Heller and Vingron 2019) in order to call SVs from the aligned PacBio HiFi, ONT, and UL-ONT reads at the different coverage levels.

The commands used for each caller and technology are listed in the Supplemental Material.

In addition, we filtered the cuteSV calls based on the minimum read support reported in the output VCF, as it generated unfiltered calls. Similarly, we filtered the SVIM calls based on the reported quality. In both cases, we used value 2 for coverages ≤5; 3 for coverages ≤10; 4 for coverages ≤20; 5 for coverages ≤25; and 10 for coverages >30. These values were selected such that they result in the highest F-scores when comparing the filtered calls to those SVs for GIAB medically relevant genes for HG002. The pipeline used for SV calling with cuteSV, Sniffles2, and SVIM can be found at GitHub (https://github.com/eblerjana/lrs-sv-calling).

Excluded regions for DELLY can be found at GitHub (https://github.com/dellytools/delly/blob/main/excludeTemplates/human.hg38.excl.tsv).

### Assembly-based variant calls

PAV (Ebert et al. 2021) was applied to phased assemblies using default parameters. Briefly, assemblies were mapped to the GRCh38 reference genome with minimap2 2.17 (Li 2018), alignment trimming was performed to eliminate redundantly mapped bases, and variant calling was performed to detect variants within alignments as well as large SVs that fragmented alignment records into multiple parts.

### Variant merging and annotations

Variant call comparisons for SNVs and indels were performed using hap.py+vcfeval (https://github.com/Illumina/hap.py, retrieved September 2, 2023; https://github.com/RealTimeGenomics/rtg-tools, retrieved September 2, 2023) to match prior precedent of benchmarking using GIAB sets. Additionally, SVs were matched using svpop and a custom merge setting (szro-50–200), which first matches variants on ID (#CHROM-POS-SVTYPE-SVLEN), then 50% reciprocal overlap, and then finally variants of the same type that are within 200 bp of each other and have reciprocal size overlap of 50%. This strategy allows for increased accuracy in complex regions of the genome where alignments can be biologically ambiguous.

Sequence content (e.g., homopolymer, tandem repeats), BED files for SNVs, and indels are based on GIAB benchmarking files available from GitHub (https://github.com/genome-in-a-bottle/genome-stratifications). Reference-based annotations for genomic sequence content for SVs are taken directly from the UCSC Genome Browser and the UCSC GoldenPath.

### F_1_ score

F_1_ score is defined as the harmonic mean between precision and recall and seeks to represent precision and recall in one metric.

$$F_{1}=2\times (\mathrm{PRECISION}\times \mathrm{RECALL})/(\mathrm{PRECISION}+\mathrm{RECALL}).$$

## Data access

HG00733 HiFi, ONT, and UL-ONT data generated in this study have been submitted to the NCBI BioProject database (https://www.ncbi.nlm.nih.gov/bioproject/) under accession number PRJNA966152.

## Supplementary Material

Supplement 1

Supplement 2

Supplement 3

Supplement 4

Supplement 5

Supplement 6

Supplement 7

Supplement 8

Supplement 9

Supplement 10

Supplement 11

Supplement 12

Supplement 13

Supplement 14

Supplement 15
