# Supplementary material for: Whole-genome long-read sequencing downsampling and its effect on variant-calling precision and recall
Source: Genome Res. 2023 Dec;33(12):2029–40. doi: 10.1101/gr.278070.123 (PMC10760522; doi:10.1101/gr.278070.123)
Supplement: Supplement 2 [file Supplemental_Material.pdf]

Supplemental Material for:

Whole-genome long-read sequencing downsampling and its effect on variant-calling precision and recall

Table of Contents

Supplemental Analyses
Supplemental Methods
Supplemental Figures S1-S12 (see attached files)
Supplemental Tables S1-S13 (see attached Excel file)

SUPPLEMENTAL ANALYSES

Assembly Contiguity

We also assessed assembly contiguity using two high-level metrics: N50 and area under the NGx curve (AuN). AuN can be defined as the expected contig length from random sampling and is meant to less directly reward assemblies that contain a few large contigs and a large number of highly fragmented contigs as opposed to N50, which is driven by the largest contigs. Unsurprisingly, input read length drives contiguity at lower coverages with UL-ONT assemblies performing the best, followed by standard ONT, and finally HiFi below 25× (**Supplemental Figure S9**). However, ONT assembly contiguity appears to reach a maximum around 15×. For assemblies generated from 25× or 30× input coverage, HiFi assemblies begin to outperform their ONT counterparts. This can be demonstrated by aligning assemblies at distinct coverages against GRCh38 (**Supplemental Figure S10**). At 10×, the increased contiguity of assemblies with UL-ONT (25.04 Mbp N50) is clearly evident compared to the 10× HiFi assembly, which has an N50 of only 250 kbp. While the difference at 30× is more subtle, the resulting N50 for trio-binned hifiasm is 50 Mbp compared to 33.63 Mbp for Flye with UL-ONT. It should be noted that haplotype phasing of ONT assemblies using HapDup had little to no effect on either N50 or AuN. This is in line with previous expectations and is due to incomplete resolution of the initial squashed assemblies (Kolmogorov et al., 2019; Shafin et al., 2020). While contiguity in ONT and UL-ONT assemblies is higher than HiFi assemblies, previous analyses have shown that the accurate resolution of variants is better in HiFi assemblies across coverages.

Genome Phasing

Long-read assemblies can be supplemented with short-read data to provide more accurate phasing information and produce, in principle, chromosome-scale haplotype-resolved contigs (Cheng et al., 2021). In these situations, parental data are leveraged to determine haplotype of origin. We observed that initial assembly quality impacts the ability of current algorithms to accurately assess haplotype of origin. Using Strand-seq-based phasing (Porubsky et al., 2021) to determine a ground truth for chromosome-scale phasing, we observe a significant increase in both switch error rate and Hamming distance in low-coverage assemblies. This trend is most significant with the 5× assembly where we observe a switch error rate and Hamming distance of 2.1 and 4.5, respectively. Above this coverage, these rates fall off greatly to 0.56 and 1, respectively, in the 8× assemblies (**Supplemental Figure S11**). Observed limits of these values

for high depth (30×) assemblies are 0.009 and 0.012. While low-coverage assemblies have respectable performance in calling variants, downstream analysis involving linkage disequilibrium and population-stratified events will be diminished.

Ti/Tv Ratio

The ratio of transitions (Ti) to transversions (Tv) is often used as a measure of SNV accuracy (Lin et al., 2022). Transitions occur at approximately twice the rate of transversions (D.W. Collins & Jukes, 1994) and our SNV truth sets reflect that rate. Read-based callers show little variability as a function of coverage, with a mean and standard deviation of 2.02 and 0.27, respectively (**Supplemental Figure S12**). In contrast, assembly-based callers, especially those using ONT reads, show greater variation across coverages with a mean and standard deviation of 2.10 and 0.26, respectively. While assembly-based methods approach a Ti/Tv rate of 2 at 30× coverage, their variability profiles are different between technologies at lower (≤15×) depths. PacBio assemblies yield a mean Ti/Tv rate of 1.87 at these coverages with ONT and UL-ONT registering a Ti/Tv ratio of 2.46 and 2.57, respectively, across both HG002 and HG00733.

SUPPLEMENTAL METHODS

Whole-genome Alignment Commands:

ONT:

```
minimap2 -ax map-ont --MD --secondary=no --eqx -x -I 8G {input.ref} {input.read}
```

PacBio:

```
minimap2 -ax map-pb -I 8G {input.ref} {input.read}
```

Read-based Variant Calling:

Clair3:

(PacBio HiFi)

```
```

run_clair3.sh --bam_fn={input.merged_bam} --sample_name={sample} --
ref_fn={input.ref} --threads={threads} --platform=hifi --
model_path=$(dirname $(which run_clair3.sh))/models/hifi --
output=$(dirname {output.vcf}) --enable_phasing
```

(ONT|UL-ONT)

```
run_clair3.sh --bam_fn={input.merged_bam} --sample_name={sample} --
ref_fn={input.ref} --threads={threads} --platform=ont --
model_path=$(dirname $( which run_clair3.sh ) )/models/ont_guppy5 --
output=$(dirname {output.vcf}) --enable_phasing
```

cuteSV:

(PacBio HiFi)

```
cuteSV -t {threads} -S {sample} --max_cluster_bias_INS 1000 --
diff_ratio_merging_INS 0.9 --max_cluster_bias_DEL 1000 --
diff_ratio_merging_DEL 0.5 {input.reference} {output.vcf} --genotype
-l 50 -s {params.min_supp} {params.outputdir}
```

(ONT|UL-ONT)

```

```
cuteSV -t {threads} -S {sample} --max_cluster_bias_INS 100 --
diff_ratio_merging_INS 0.3 --max_cluster_bias_DEL 100 --
diff_ratio_merging_DEL 0.3 {input.reference} {output.vcf} --genotype
-l 50 -s {params.min_supp} {params.outputdir}
```

DeepVariant:

(PacBio HiFi)

```
run_deepvariant --model_type=PACBIO --ref={ref} --reads={aln} --
output_vcf={sample}.vcf.gz --output_gvcf={sample}.gvcf --
novcf_stats_report --intermediate_results_dir=/dv_tmp/ --
num_shards={threads}
```

(ONT-duplex)

```
run_deepvariant --model_type=ONT_R10 --ref={ref} --reads={aln} --
output_vcf={sample}.vcf.gz --output_gvcf={sample}.gvcf --
novcf_stats_report --intermediate_results_dir=/dv_tmp/ --
num_shards={threads}
```

DELLY:

(PacBio HiFi)

```
delly lr -y pb -g {input.ref} -x {input.exc} -o {output.bcf} {input.bam}
```

```

(ONT|UL-ONT)

```
delly lr -y ont -g {input.ref} -x {input.exc} -o {output.bcf} {input.bam}
```

PBSV:

(PacBio HiFi)

```
pbsv discover --tandem-repeats {input.trf} {input.bam} {output.svsig}
pbsv call -j {threads} --ccs --types DEL,INS,INV {input.ref} {input.svsig}
{output.vcf}
```

PEPPER-Margin-DeepVariant:

(ONT|UL-ONT)

```
run_pepper_margin_deepvariant call_variant -b {bam} -f {ref} -o {out_dir} -
t {threads} --ont_r9_guppy5_sup
```

Sniffles:

(PacBio HiFi|ONT|UL-ONT)

```
sniffles -t {threads} -i {input.bam} -v {output} --reference {input.reference} -
-minsvlen 50
```
```

SVIM:

(PacBio HiFi|ONT|UL-ONT)

```
svim alignment {params.outdir} {input.bam} {input.reference} --
min_sv_size 50
```
